# Comparative Analysis of Yeast Metabolic Network Models Highlights Progress, Opportunities for Metabolic Reconstruction

**DOI:** 10.1371/journal.pcbi.1004530

**Published:** 2015-11-13

**Authors:** Benjamin D. Heavner, Nathan D. Price

**Affiliations:** Institute for Systems Biology, Seattle, Washington, United States of America; Hellas, GREECE

## Abstract

We have compared 12 genome-scale models of the *Saccharomyces cerevisiae* metabolic network published since 2003 to evaluate progress in reconstruction of the yeast metabolic network. We compared the genomic coverage, overlap of annotated metabolites, predictive ability for single gene essentiality with a selection of model parameters, and biomass production predictions in simulated nutrient-limited conditions. We have also compared pairwise gene knockout essentiality predictions for 10 of these models. We found that varying approaches to model scope and annotation reflected the involvement of multiple research groups in model development; that single-gene essentiality predictions were affected by simulated medium, objective function, and the reference list of essential genes; and that predictive ability for single-gene essentiality did not correlate well with predictive ability for our reference list of synthetic lethal gene interactions (R = 0.159). We conclude that the reconstruction of the yeast metabolic network is indeed gradually improving through the iterative process of model development, and there remains great opportunity for advancing our understanding of biology through continued efforts to reconstruct the full biochemical reaction network that constitutes yeast metabolism. Additionally, we suggest that there is opportunity for refining the process of deriving a metabolic model from a metabolic network reconstruction to facilitate mechanistic investigation and discovery. This comparative study lays the groundwork for developing improved tools and formalized methods to quantitatively assess metabolic network reconstructions independently of any particular model application, which will facilitate ongoing efforts to advance our understanding of the relationship between genotype and cellular phenotype.

## Author Summary

Scientists have been mapping the chemical reactions cells use to grow and manage waste since before enzymes were first identified more than 150 years ago. The model yeast *Saccharomyces cerevisiae* has one of the most extensively studied metabolic networks, including at least 25 metabolic network models published since 2003. If iterative model improvement refines the metabolic network map, we would expect eventual convergence to a full, accurate metabolic network reconstruction. In this study, we looked for evidence of such convergence through comparative analysis of 12 genome-scale yeast models. We conducted simulations and evaluated model features such as predictive accuracy, genomic coverage and the included metabolites and reactions. We found that no single metric for evaluating models can adequately summarize important aspects of model quality. In some cases, we observed tradeoffs between model predictive accuracy and network coverage. We found evidence of incremental changes to the network reconstruction, but not marked shifts in model predictive ability or other metrics clearly arising from changes to the network alone. This work has broader implications to computational reconstruction of metabolic networks for any organism, and suggests that there is opportunity for refocusing the model building process to better support mapping cellular metabolic networks.

## Introduction

Efforts to map metabolic networks—to describe the full network of anabolic and catabolic biochemical reactions occurring within a cell—have advanced from early biochemical studies of fermentation [1] to contemporary efforts to algorithmically generate pathway diagrams from genomic sequence [2]. Such pathway maps may be augmented with additional metadata to build a digital “reconstruction” of an organism’s metabolic network. In turn, such organism-specific reconstructed metabolic networks may be further supplemented to build mathematical models that are capable of simulating metabolic fluxes [3]. Recently, research efforts have focused on improving the ability to quickly build genome-scale metabolic network models of metabolism and to improve their predictive accuracy [2,4–6]. Comparatively less effort has been spent exploring opportunities for knowledge discovery arising during the process of network reconstruction prior to mathematical simulation [7]. In this work, we emphasize the distinction between metabolic network “reconstruction” and metabolic network “model”. Emphasizing this distinction facilitates an effort to resolve the relative contributions to model predictive accuracy or error arising from the metabolic network structure itself (the “reconstruction”) from those arising from mathematical parameters chosen when building a simulatable metabolic network “model” from the network reconstruction. While a variety of *ad hoc* quantitative metrics have been applied to evaluate improvements in metabolic network models, quantified assessment of the progress of the underlying reconstructions over time is a nascent effort [8]. This may be, in part, due to the fact that the number of models is so much greater than the number of extensively curated reconstructions. The relative difficulty of curating a comprehensive metabolic network reconstruction compared to generating a draft model is highlighted by the fact that there are currently more than 2,600 functional draft models [6], but only Escherichia coli [9] and Saccharomyces cerevisiae [10] reconstructions have been extensively updated multiple times and revised through curation efforts by multiple research groups over a multi-decade timescale. Since simulation results are more amenable to quantitative analysis than reconstruction quality, reconstructions have generally been assessed indirectly, often in the context of model performance via manuscript discussion of scope (the number of genes, reactions, or metabolites in a model), standards compliance, naming or annotation conventions, reputation of the group that built a particular model, or predictive performance of a derived model for a particular phenotype of interest (commonly used phenotypes include gene essentiality, substrate utilization, growth rate, or product production) [11]. This indirect approach to assessing metabolic network reconstruction quality bears a risk, however, because the model building process itself can obscure important details about the underlying reconstruction (particularly knowledge limitations that may be useful for informing future investigation [12,13]).

The standard reconstruction protocol includes converting a reconstruction to a mathematical model for subsequent debugging [3]. Thus, the ability to create a functional model has come to serve as a minimum threshold for defining the scope of a “draft reconstruction”, and the distinction between “reconstruction” and mathematical “model” has become blurred. Model developers are free to take different approaches when parameterizing model features such as objective function (i.e., biomass composition) [14,15], media definition [16], and reference lists of “essential” genes used for benchmarking model performance [17]. Model developers may use different approaches to gap-filling [4,18,19], trimming dead-end metabolites, establishing an objective function, and adding transport and exchange reactions. In fact, optimization-based approaches have been applied to successfully improve model essentiality predictions by adjusting these parameters [20]. Such algorithmic approaches can improve the predictive performance of a model even in the absence of any changes to the underlying metabolic reconstruction.

Using model performance to drive iterative improvements to metabolic network reconstruction has led to two perverse consequences. First, if two models of the same organism give different predictions, how can a researcher determine whether the differences arise from differences in the reconstructed network or from differences in model parameters? We have previously observed that algorithms such as OptKnock [21] can suggest different targets for metabolic engineering efforts when applied to different models of the same organism (unpublished data). Second, a single metabolic network model can provide only limited information about the quality of the underlying metabolic network reconstruction because there are so many degrees of freedom associated with deriving a model from a reconstruction [7,8]. Comparative analysis of multiple models, now possible at scales not previously feasible [22], provides an opportunity to address these challenges of single-model analysis. Our approach is to conduct comparative analysis of yeast metabolic network models that have been published in the past two decades, while controlling for differing modeling assumptions with a standardized model biomass function, media definition, and common sets of genes considered in the evaluations. An additional benefit of comparative analysis of models spanning a multi-decade timescale is the opportunity for evaluating model predictive performance on data that was not available at the time of reconstruction, which can provide a useful independent validation procedure and provide insights into the degree of overfitting possible in these models through the manual reconstruction process that is very difficult to ascertain otherwise.

At least 25 models of the *Saccharomyces cerevisiae* metabolic network have been published since 2003 [5,11,17,20,23–40]. Each of these models has been applied successfully to research efforts focusing on advancing biotechnology [41], mapping genotype to phenotype relationships in cellular physiology [42], or developing new methods in computational biology [43]. Previously, researchers have combined comparative analysis of three of these models (iFF708, iLL672, and iND750) with experimental data to refine characterization of cellular phenotypes in 16 environmental conditions [44], and developed tools to facilitate model matching and comparison for synchronous investigation or building composite models [45]. Another two models (Yeast 5 and iMM904) have been evaluated for predicting growth rates of a prototrophic gene deletion library in 20 different conditions [46]. More recent efforts have begun comparative analysis of a broader range of these models [22]; however, we are not aware of previous large-scale comparative analysis efforts that modify model objective functions, reference phenotype lists, and simulated media composition to evaluate the underlying metabolic network reconstructions built for *S*. *cerevisiae*.

In this study, we conducted 161 in silico screens of predicted single gene essentiality using 18 different simulated media conditions, 12 different yeast metabolic models, and 13 different biomass definitions. We used this range of simulation parameters to standardize choices made in the development of various models, thus facilitating evaluation of the underlying network reconstructions. Using a binary growth/no growth assessment metric, we evaluated model predictions of the essentiality of three different lists of “essential” genes compiled from literature and database review. Additionally, we conducted simulations of aerobic growth with constraints on glucose, oxygen, and nitrogen exchange reactions singly or in combination to evaluate the correlation between model predictions of maximally achievable biomass flux values and reported experimental growth rates. We also conducted 10 in silico screens of pairwise gene essentiality by different models using their default media and biomass definitions, and compared model predictions to 32,488 gene pairs annotated as synthetic lethal in the Saccharomyces Genome Database. All code for our analysis is available as S1 File. Our key findings include the following. (1) Changes in model scope reflect a history of iterative reconstruction development via collaboration between groups—in other words, each model contains evidence of its history, with stylistic and content evidence of the specific model from which it is derived. Knowledge is propagated between models, but there is also risk of error propagation. Therefore, it is important to revisit assumptions made when earlier models were originally built when evaluating newer models. (2) Model updates tended to fall into two major categories, model scope expansion (i.e. the inclusion of new metabolic processes) or subsequent refinement (i.e. including essentially the same sets of processes but working to improve accuracy). There was a pattern in analyzing the models’ ability to predict KO essentiality that accuracy on average reduced when model scope expansion was done and then improved on subsequent reconstructions aimed at improving the same set of processes. (3) For each model, single-gene essentiality predictions were affected by parameters external to metabolic network structure, such as simulated medium, objective function, and the reference list of essential genes. (4) The correlation between model predictions of maximum biomass flux correlate and reported growth rates are the same for all models when only a single exchange reaction is constrained, but the correlation between model prediction and reported growth rate differ among the models when multiple exchange reactions are simultaneously constrained to experimental values. This difference can be attributed to changes in metabolic network reconstructions independent of model parameters. (5) The predictive ability for single-gene essentiality did not correlate with predictive ability for our reference list of pairwise synthetic lethal genes. Thus, we conclude that the reconstruction of the yeast metabolic network is generally improving, and have demonstrated that comparative model analysis contributes to reconstruction improvement. There remains great opportunity for advancing our understanding of metabolic function through continued efforts to improve the reconstruction of the yeast metabolic network.

## Results

### 1) Changes in model scope reflect the history of model development and collaboration between groups

**Fig 1:**
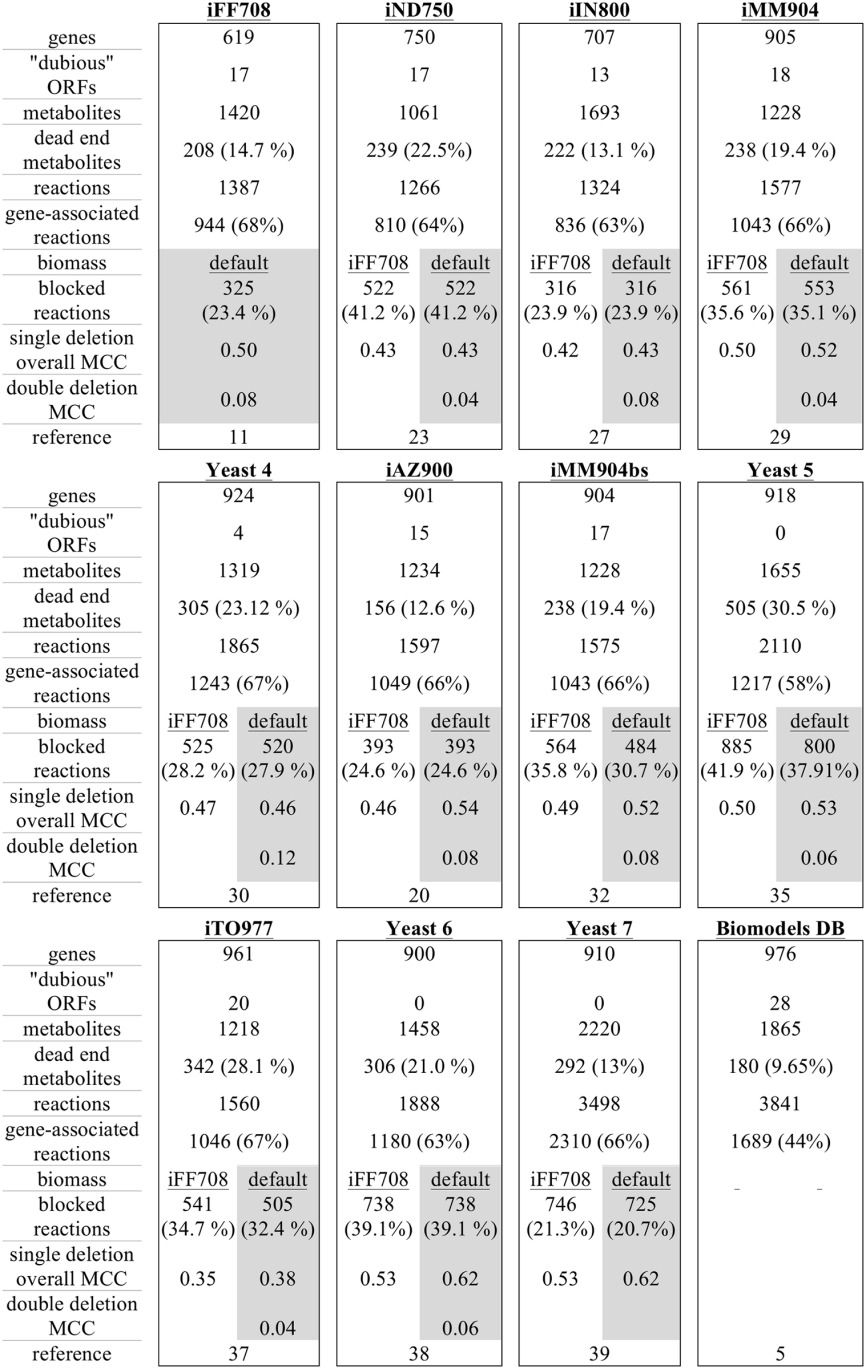
Summary statistics of yeast metabolic network models. General parameters described or used for simulation in this study include: number of genes included as reaction modifiers; number of included genes that are currently annotated by the Saccharomyces Genome Database as “dubious”, or unlikely to encode an expressed protein; number of metabolites; number of dead end metabolites (those metabolites that are either produced by known metabolic reactions of an organism but not consumed, or vice versa); number of reactions; and number of reactions associated with genes. Simulations were conducted for each model using the as-distributed model default biomass objective function and with the biomass objective from the iFF708 model. Reported simulation results are divided into two subcolumns to reflect the use of two different objective functions for each model. Simulation results include the number of blocked reactions for each biomass definition (those reactions which cannot carry flux due to network structural constraints); the Matthews’ Correlation Coefficient (MCC) for model predictions of single gene essentiality across all conditions simulated; and the Matthews’ Correlation Coefficient for model prediction of double gene essentiality (i.e., pairwise synthetic lethal interactions) for simulations using each models’ default biomass definition. Some parameter values differ from previously published values due to differing software implementation and annotation conventions.

We compiled summary statistics for functional yeast metabolic models published since 2003 (Fig 1), including the number of metabolites, reactions, dead-ends, gene-associated reactions, and genomic coverage. When the models are ordered chronologically, none of these statistics demonstrates continuous improvement, perhaps reflecting the differing research objectives that motivated the development of each new model, but also demonstrating the limited ability of any individual statistic for fully describing model quality (e.g. reducing the number of genes in a reconstruction is an improvement if previous iterations included misannotated genes, but such a reduction of genomic coverage could be considered a worse statistic).

We observed a general increase in the number of genes included in models over time, but this increase was not uniform. We also found that increased genomic coverage could be a result of modelers including genomic features that are no longer considered genes. For example, the Biomodels.db model accounts for the greatest coverage of the yeast genome, but includes 28 open reading frames currently annotated as “dubious”, or unlikely to encode a functional protein. Additionally, increases in genomic coverage did not imply improved predictive accuracy: Yeast 6 includes fewer genes than Yeast 5, but improves single-gene essentiality predictions

Similarly, the number of metabolites and reactions and the proportion of dead-end metabolites in yeast metabolic models has generally, but not uniformly, increased over time—and does not coincide with improved predictive ability. For example, the number of metabolites and reactions in Yeast 7 is much larger than that in Yeast 6, though they have the same overall MCC. In contrast, iND750 contains fewer metabolites and reactions than its progenitor model iFF708. The portion of dead-end metabolites—those metabolites that are consumed but not produced in the network or vice versa—has also varied among models, and does not correlate with predictive accuracy.

**Fig 2:**
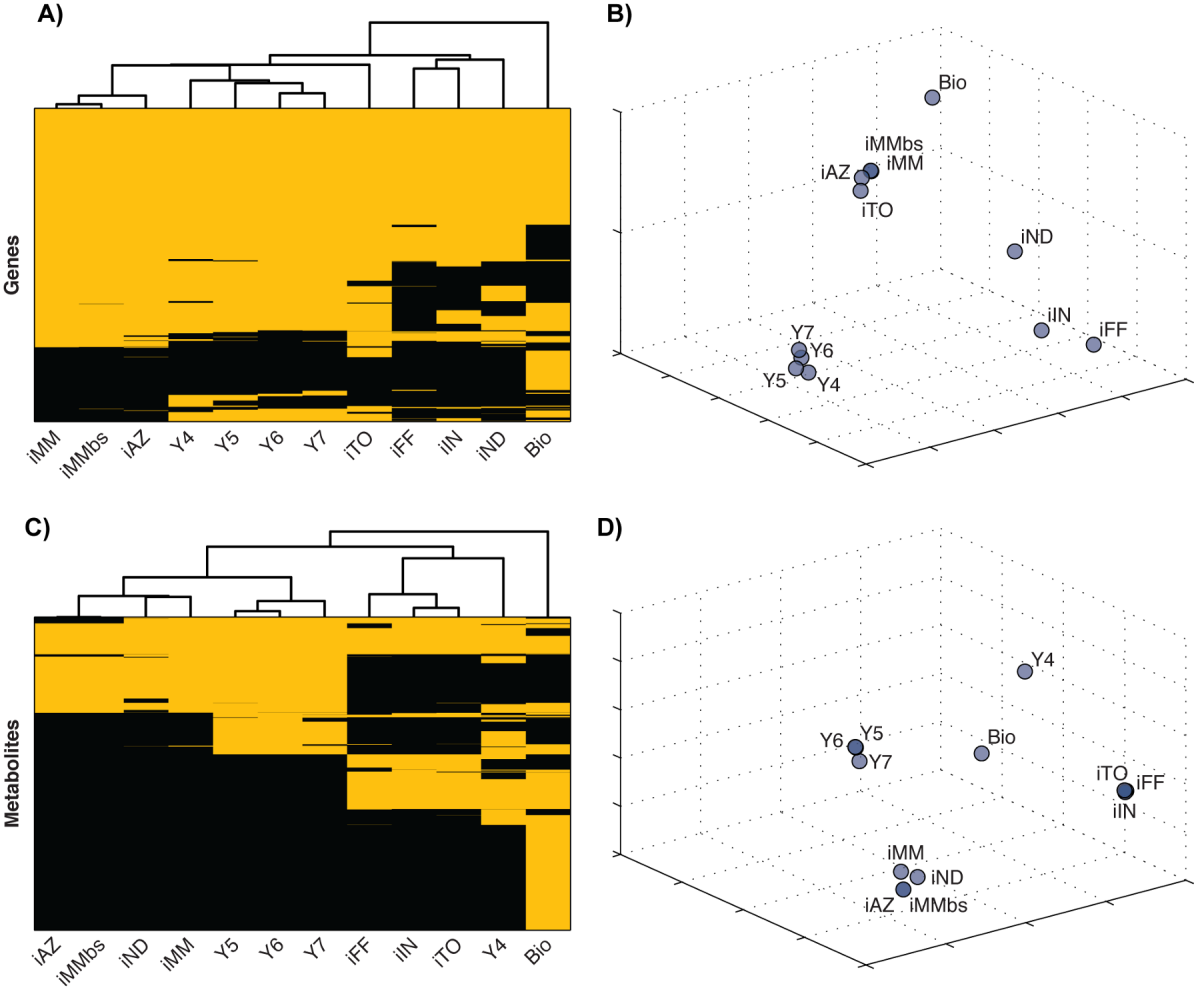
Comparing model genomic coverage and metabolite annotation. Models clustered differently when compared using genomic coverage (A and B) or the subset of metabolites in each model that are annotated with reference to an external database such as the Chemical Entities of Biological Interest (ChEBI) database (C and D). Results are presented as heatmaps with dendrograms (A and C) and scatterplots of the normalized pairwise distance between models (B and D). Yellow bands in the heat map signify inclusion of a particular open reading frame in that model, and dendrogram clustering is based upon similarity.

Next, we evaluated model scope by comparing genomic coverage and the metabolites that could be cross-identified with Chemical Entities of Biological Interest (ChEBI) identifiers with the annotation included with the models. We were unable to directly compare model reactions because of the lack of standardized reaction identification between models, and the lack of an external reaction reference database identifier in any yeast metabolic model, a current limitation for interoperability and comparison in our field. We found that models clustered in groups that reflect their historical development [10], but these clusters differ between gene and metabolite comparisons (Fig 2). Models clustered in 4 groups when comparing genomic coverage: 1) Versions 4–7 of the Consensus Reconstruction; 2) iMM904, iMM904bs, iAZ900, and iTO977; 3) a looser cluster of iFF708, iIN800, and iND750; and 4) the Biomodels.db model. A row-aligned comparative table of genes in each model is included as S1 Table.

Model similarity clusters differed when based upon ChEBI identifier-annotated metabolites, and the clusters were more tightly linked to the research group most closely related to the development of a group of models. When clustered by annotated metabolites, the resulting 5 groupings consisted of 1) Versions 5–7 of the Consensus Reconstruction; 2) iND750, iMM904, iMM904bs, and iAZ900; 3) iFF708, iIN800, and iTO977; 4) Version 4 of the Consensus Reconstruction and 5) the Biomodels.db model.

### 2) Single-gene essentiality predictions reflect the iterative process of model scope expansion and subsequent refinement

**Fig 3:**
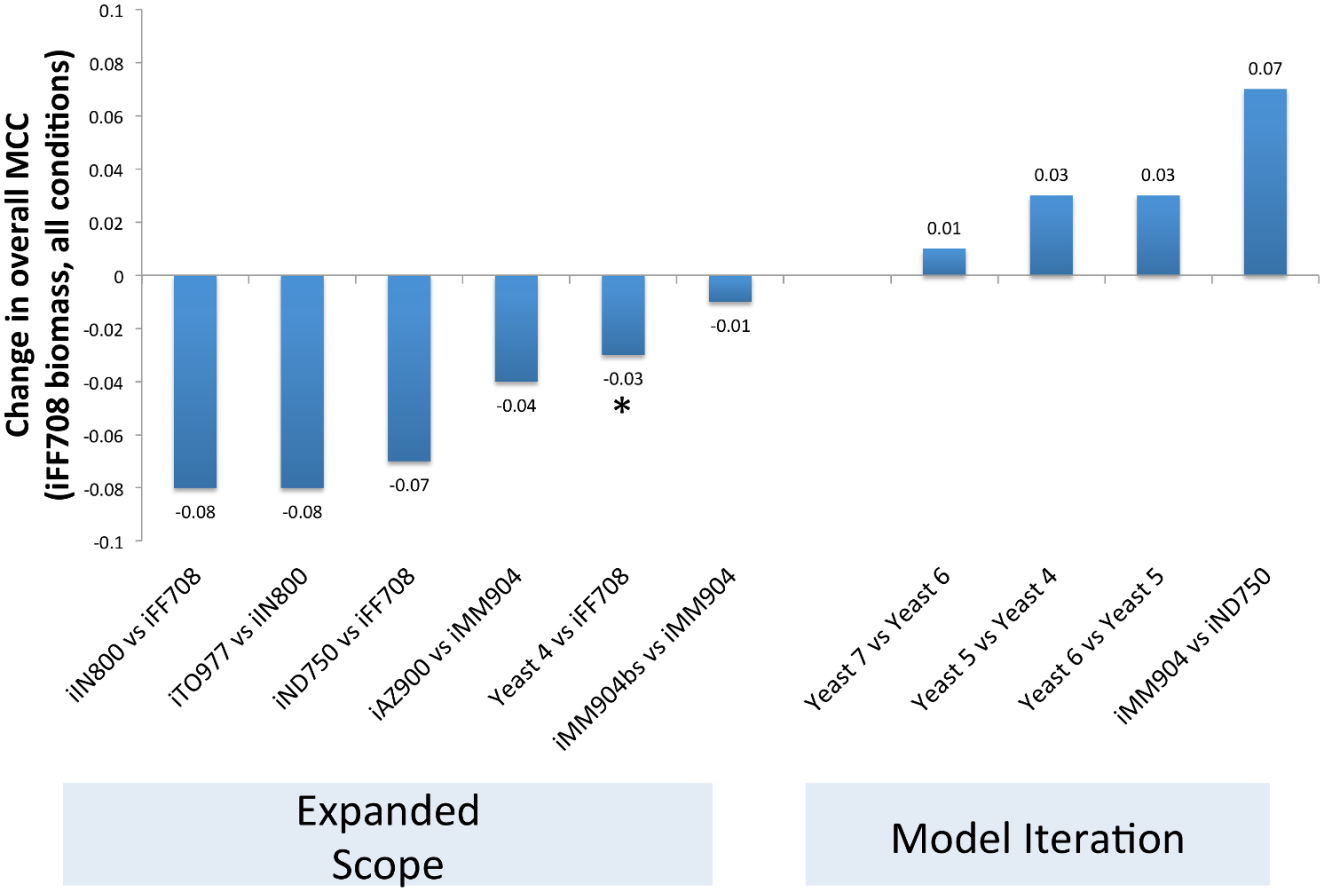
Change in gene essentiality predictions between model and its nearest ancestor. When comparing the Matthews Correlation Coefficient for model gene essentiality predictions to the models’ nearest progenitors, we observe that the models may be segregated between those focusing on expanding model scope, and those focused on iterative refining an existing model by plotting the change in MCC between models. Generally, when the stated focus of a model developer is to expand the scope of the yeast metabolic network reconstruction, predictive ability suffers relative to the progenitor model. When the stated focus is to refine and curate a model, predictive ability improves relative to the progenitor model. Thus, our analysis finds that model predictive ability reflects the iterative process of model development. The asterisk near the Yeast 4 comparison indicates that it is an integrative model that not have a single nearest progenitor (we compared it to iFF708 for this analysis).

Improving model ability to predict the essentiality of individual genes for growth has not been the primary motivating factor for developing each new yeast metabolic network model, but essentiality predictions have generally been reported with the publication of each new model to demonstrate their accuracy and utility. However, direct comparison between reported predictive values is complicated by differing simulation conditions. In this study, we did not find a strong trend of continuing improvement in model ability to predict single gene essentiality over time. Instead, we found evidence of an iterative process in which model scope changes (typically leading to a decrease in average predictive accuracy), followed by subsequent curation leading to improved prediction by descendant models (Fig 3). We found that both iIN800, with its expansion of the reconstruction of lipid metabolism, and iND750, with its expansion of compartmentalization, had a lower overall Matthews Correlation Coefficient (MCC) for single-gene essentiality predictions than their progenitor model, iFF708. Subsequently, iMM904 refined iND750, and made more correct predictions of single gene essentiality. Similarly, Yeast 6 refines Yeast 5 and improves predictive ability, but the focus of Yeast 7 on expanded scope does not lead to as large an improvement in single-gene essentiality predictive ability, and iTO977’s focus on expanding model scope to cover some protein modification processes and to provide a scaffold for integrating transcriptomic data does not lead to an improvement in predicting single-gene essentiality compared to its progenitor model, iIN800.

The Biomodels.db model was generated in a methods-development effort to improve automated reconstruction and annotation. The algorithm underlying the Biomodels.db model prioritizes connectivity and defines “functionality” as the ability of the model to predict growth using a generic biomass definition objective function. The Biomodels.db annotates genes with a different nomenclature than the ORF format used by other models, so was incompatible with our gene essentiality screen. We evaluated the Biomodels.db model by other comparative metrics, but did not evaluate its FBA performance here.

### 3) For each model, single-gene essentiality predictions were affected by simulated medium, objective function, and the reference list of essential genes

**Fig 4:**
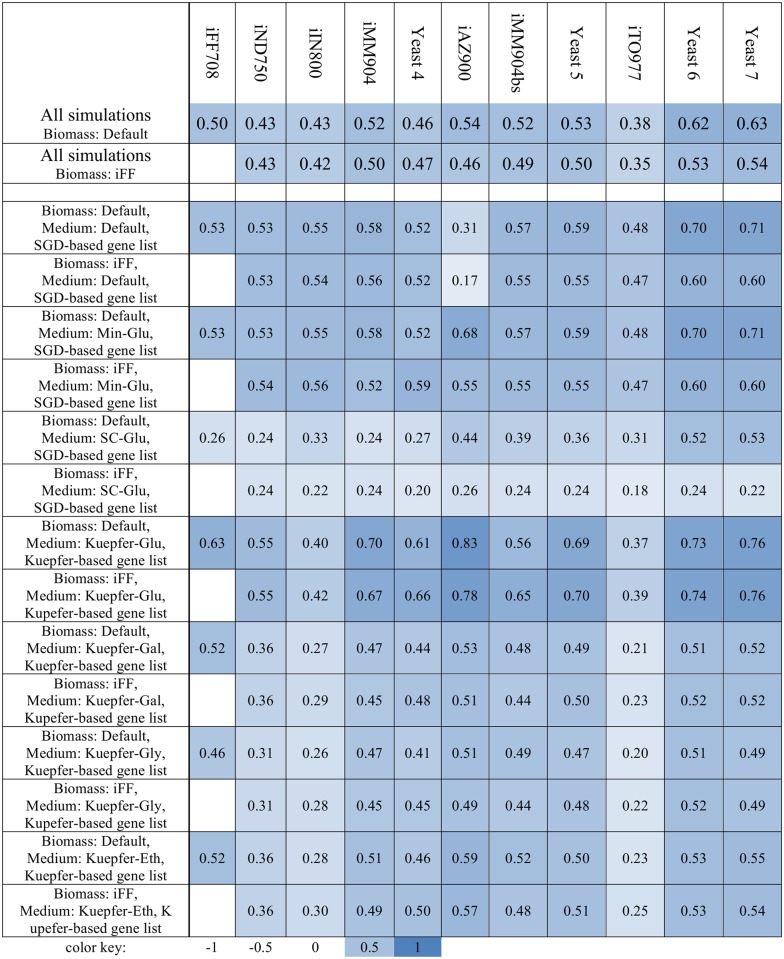
Evaluating model predictions of single-gene essentiality. Flux balance analysis was conducted to predict whether individual genes were essential for growth using seven different media formulations and two different model biomass objective functions for each model. Gene essentiality predictive performance is summarized in this table by the Matthews’ Correlation Coefficient (MCC). Model predictions were compared to two reference lists of essential genes: one derived from the saccharomyces genome database (SGD-based gene list) and one from Kuepfer et al. (Kuepfer-based gene list). These lists are provided as Supplementary Information. Modeled medium formulations included each model’s default medium, (Medium: Default), a minimal glucose-limited medium (Medium: Min-Glu), a synthetic complete glucose-limited medium based on Kennedy et al. (Medium: SC-Glu), and a synthetic medium based on Kuepfer et al. using glucose (Medium: Kuepfer-Glu), galactose (Medium: Kuepfer-Gal), glycerol (Medium: Kuepfer-Gly), or ethanol (Medium: Kuepfer-Eth) as carbon source. Simulations were conducted using each model’s default biomass definition (Biomass: Default) or the iFF708 model biomass definition (Biomass: iFF). In this heat map, color intensity is based upon positive Matthews’ Correlation Coefficient (MCC) (no parameter combinations lead to negative MCCs for any model), each row is a unique set of model parameters, and models are arranged in chronological order from left to right.

We conducted 161 simulated genome-wide single gene deletion screens for gene essentiality by conducting flux balance analysis with an objective of maximizing biomass flux. We used different combinations of simulated media and biomass objective functions and compared model predictions to appropriate reference lists of essential genes, as described in the Materials and Methods section. We found that no single model predicted essential genes best in all simulations (Fig 4). In our simulations, the iAZ900 model had the highest single MCC we calculated (0.83) for a particular condition, and the Yeast 7 model had the highest overall MCC across all the conditions for which we calculated (0.61). As a point of comparison, we calculated a MCC of 0.61 based on the reported results of a gene essentiality screen with a recent model of the *E*. *coli* metabolic network [47], which is, along with yeast, widely considered the best studied genome-scale metabolic network model to date.

Although it had the highest observed MCC in one condition, the iAZ900 model did not perform as well in other simulations—it also had the lowest MCC (0.17) for an out-of-sample screen using the iFF708 biomass definition, a very permissive set of exchange reactions, and a reference gene list based upon SGD-reported phenotypes. When the exchange reactions are constrained to reflect a simulated glucose minimal defined media, the iAZ900 MCC for the iFF708 biomass increases to 0.55. Such ranges of model predictive ability were observed for all models across differing simulation conditions, highlighting the importance of controlling for model parameter variation when attempting to compare metabolic network models of a particular organism. In the specific case of iAZ900, the excellent performance of its best condition reflects the authors’ goal in developing iAZ900 –to use an algorithmic approach to improve the iMM904 model by maximizing agreement with a list of genes essential genes reported to be essential by Kuepfer et al. [17]. The reference list of essential genes used in the development of iAZ900 originates from a screen of non-essential genes in the yeast knockout collection in glucose-limited defined medium [48]. This reference list for training the algorithm is one of the reference lists we used for comparative evaluation. iAZ900 did not perform as well at classifying genes as essential when using other reference gene lists. Thus, iAZ900 demonstrates that high model performance can be achieved by one metric, but there is the usual tradeoff between sensitivity and specificity when attempting to generalize a specific metabolic network model to predict phenotypes in new conditions.

Our observation that model performance was influenced by the reference list of genes considered essential when attempting to evaluate model predictive ability demonstrates that the definition for gene essentiality is another parameter that may be tuned as model developers refine their model. In our simulations, model MCC was higher on average when calculated relative to the SGD-based list of essential genes for five of the models (iND750, iIN800, iTO977, Yeast 6, and Yeast 7), and higher relative to the Kuepfer-based list of essential genes for the remaining six models (iFF708, iMM904, Yeast 4, iAZ900, iMM904bs, and Yeast 5). These two groups do not correspond to the clusters identified when comparing model genomic coverage or the clusters identified when comparing annotated metabolites.

All models predicted gene essentiality better when glucose was the simulated primary carbon source than when galactose, glycerol, or ethanol were the primary sources. However, since the reference gene list used for the non-glucose carbon sources was based upon a single screen, we could not determine whether this reflects limitations in the reconstruction of the non-glucose metabolic network, or strain and laboratory-specific effects in the reference data. Historically, the metabolism of non-glucose carbon sources has received less biochemical characterization than glucose metabolism in yeast.

**Fig 5:**
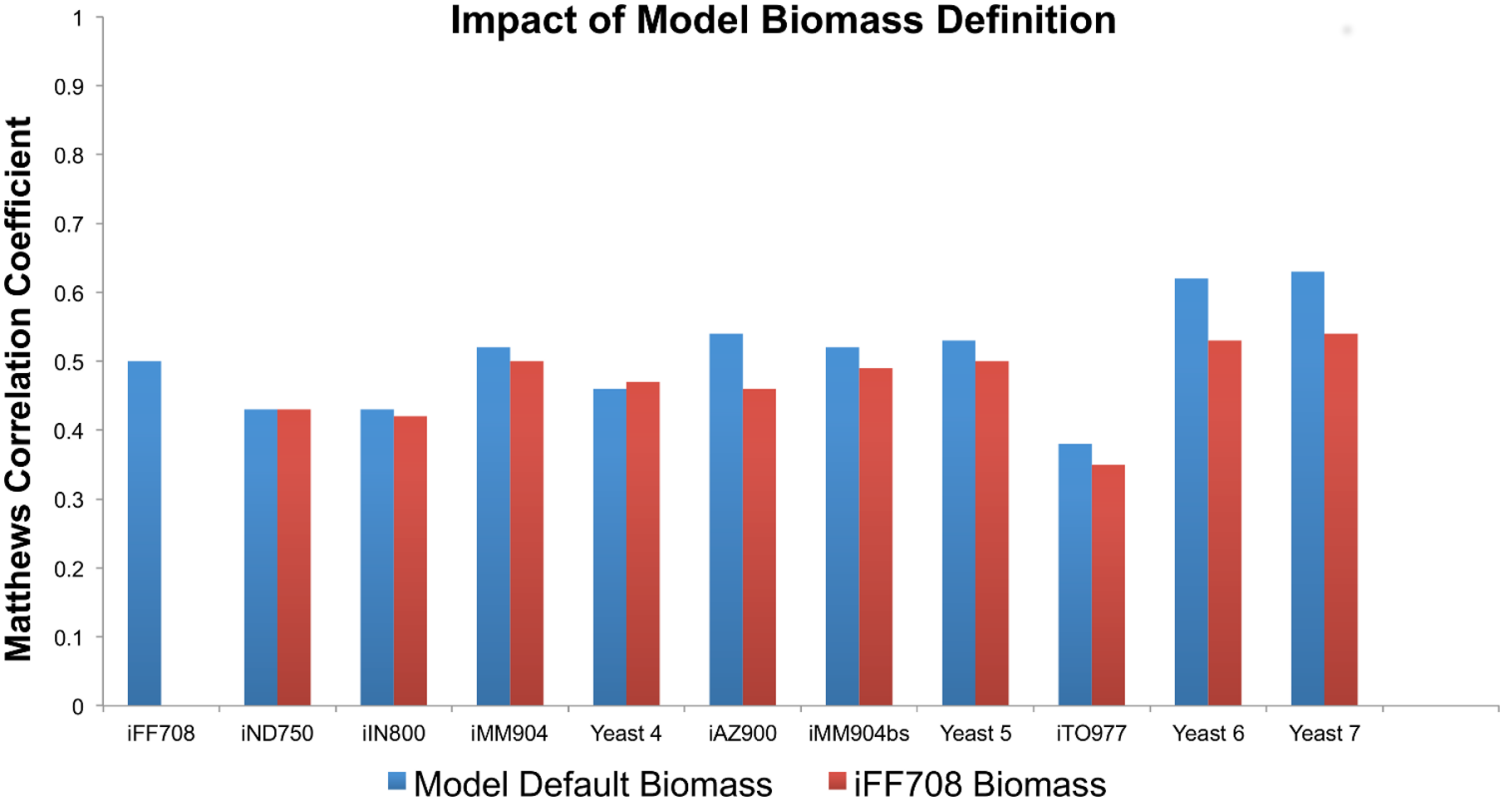
Model prediction of single-gene essentiality is sensitive to biomass definition. Since objective function is a tunable model parameter, we calculated Matthews’ Correlation Coefficients for the sum of all true positive, true negative, false positive and false negative predictions across all conditions using two different objective functions for each model: the biomass definition provided by the model authors, and the biomass function used for the iFF708 model. We found that with the exception of the Yeast 4 model, all model predictions were improved by tuned objective function, independent of refinements to the biochemical network reconstruction. Models are arranged in chronological order across the horizontal axis.

Since the objective function is a tunable parameter that is independent of metabolic network structure, we normalized the objective by selecting a biomass definition that each model could satisfy, as described in the Flux Balance Analysis—Biomass Definition subsection of Methods, below. Thus, we began differentiating between model parameter improvements and network structure improvements to compare the reconstruction underlying different models more directly. We performed Flux Balance Analysis of the metabolic network models using both the biomass definition provided by the model authors, and the biomass function used for the iFF708 model, and found that for all models with different biomass definitions than the iFF708 model, the model predictive power was affected by the objective function used (Fig 5). In every case but the Yeast 4 model, model predictions were better using the model default biomass objective than the iFF708 objective, suggesting that model developers have achieved improved predictive accuracy in part by modifying the objective function, and such improvements have been achieved independently of refinements to the biochemical network reconstruction itself. This approach is not meant to imply that modifications to an objective function would be conducted solely to improve a predictive metric: refinements to the biomass definition also reflect improved measurement of biomass composition and changes to model scope. We selected a common biomass definition for our analysis to evaluate the impact of this particular model parameter.

### 4) Model predictions of maximum biomass flux correlate with reported growth rates similarly when a single exchange reaction is constrained, but differently when multiple exchange reactions are constrained to experimental values. This difference can be attributed to changes in metabolic network reconstructions independent of model parameters

We conducted FBA-based comparison of media- and objective-normalized model predictions of maximum achievable biomass fluxes with the aerobic growth rates reported by Österlund et al. for “N-limited” and “C-limited” conditions (we did not simulate anaerobic growth since most of the models we are examining do not predict anaerobic growth on a minimal medium). We found that the model predictions of maximally achievable biomass flux correlated with the previously reported “N-limited” growth rates with a correlation of 0.994 when nitrate or nitrite exchange fluxes were constrained to previously reported uptake rates (S3 Table).

**Fig 6:**
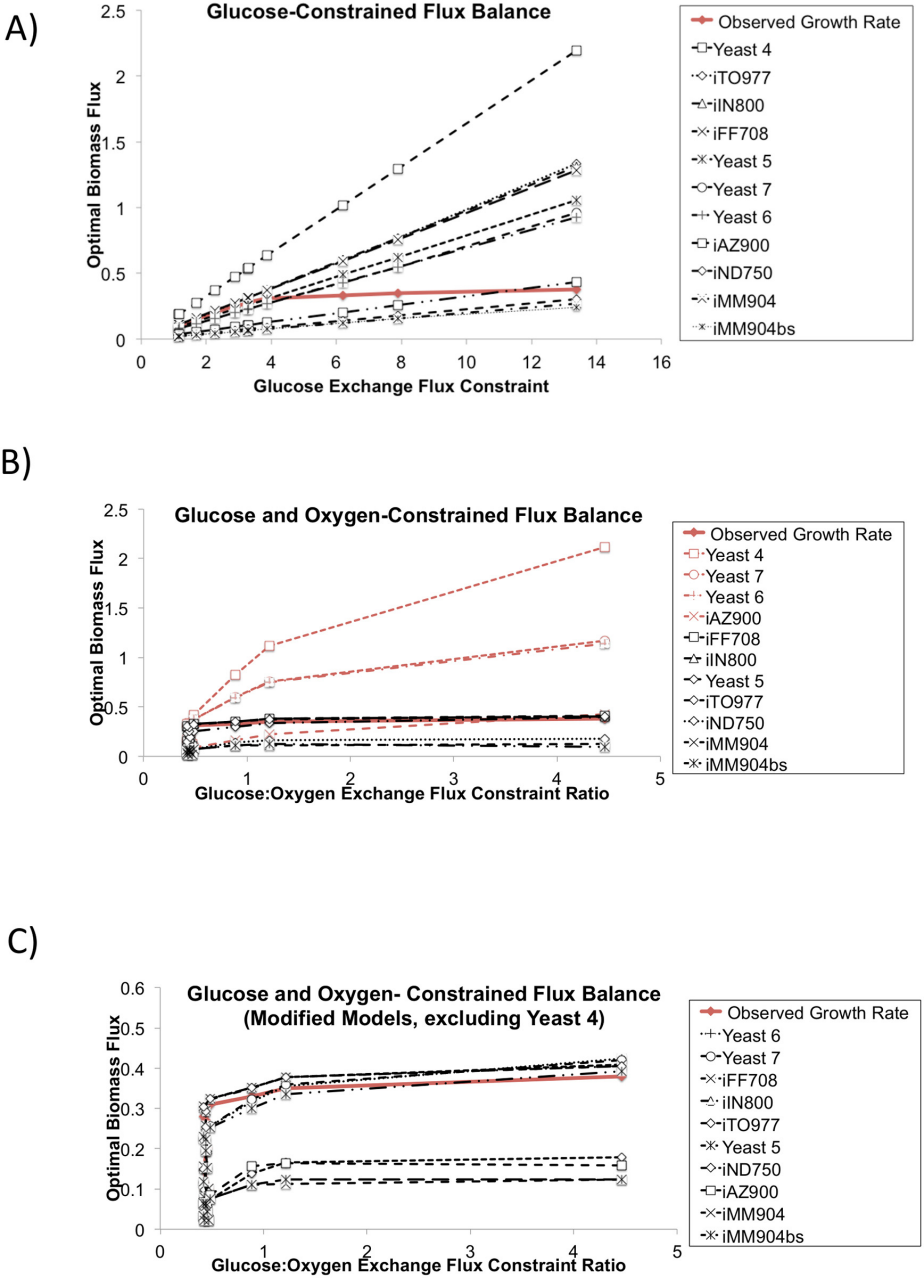
Growth simulations demonstrate interplay between network reconstruction and constraints. A) Optimal biomass flux calculated by flux balance analysis increased linearly with glucose uptake flux for all models when the glucose exchange reaction is the only constrained media component. All model predictions had a 0.8158 correlation with previously reported measured growth rate. B) When glucose and oxygen exchange reactions were both constrained to experimental values, there are high-correlation (black) and low-correlation models (red). C) Restricting flux through a mitochondrial aspartate transport reaction did not affect the predictions for the high correlation models, and improved all remaining correlations to >0.9, with the exception of the Yeast 4 model, which still over-predicted the maximum biomass flux at high glucose:oxygen exchange constraint ratios.

The “C-limited” simulations reflected a different behavior. When we constrained the glucose exchange reaction alone, all models had a 0.816 correlation with the reported growth rates (Fig 6A). However, the growth rates labeled “C-limited growth aerobic” by Österlund et al. are not linear over the range of constraints imposed on the glucose exchange reaction, suggesting that carbon (glucose) flux is not the sole growth-limiting factor, particularly at the higher range of glucose flux constraints. The ratio of glucose exchange flux to oxygen exchange flux would be expected to strongly influence maximum achievable biomass flux due to stoichiometric constraints on the oxidation of glucose [49]. We tested model behavior against this expectation by conducting FBA with both glucose and oxygen exchange reactions constrained to values reported by Österlund et al. [37]. When glucose and oxygen exchange reactions were both constrained to experimental values, we observed that the models segregated to 2 groups: biomass flux predictions made by 7 models (iFF708, iIN800, Yeast 5, iTO977, iMM904, and iMM904bs) correlated with observations with a correlation >0.9, and predictions made by the remaining models (Yeast 4, Yeast 6, Yeast 7, iAZ900) had lower correlations (Fig 6B).

We used one-norm minimized FBA [50] to find an explanation for this difference in model predictions and observed unrealistically large fluxes through internal reactions along with unusually large exchange fluxes in models that overpredict biomass flux in high glucose:oxygen growth simulations. Through repeated FBA and manual investigation of high-flux loops, we found that the low-correlation models all had a flux through a mitochondrial aspartate transport reaction. This reaction is not associated with a gene in the iAZ900 model (reaction id “ASSPt2M”), but is annotated with yeast open reading frame YPR021C in the Yeast 4, Yeast 6, and Yeast 7 models (reaction ids “r_1163”, “r_1117”, and “r_1117”, respectively). YPR021C encodes Agc1p, a protein that “fulfills two functions… glutamate transport into mitochondria … and … aspartate-glutamate exchanger within the malate-aspartate NADH shuttle” [51]. Subsequently, we also found this reaction in the iND750 (“ASPt2M”), iIN800 (“AGC1_2”), iMM904 (“ASPt2m”), iMM904bs (“ASPt2m”), Yeast 5 (“r_1117”), and iTO (“AGC1_2”) models. We did not find this reaction in iFF708 or the Biomodels.db models, which do not include mitochondria as a separate compartment. We did not find literature support for including yeast mitochondrial aspartatate transport as reconstructed in these models. Thus, investigating erroneous predictions of maximum biomass flux by four models at simulated high glucose:oxygen flux states allowed us to identify a reconstruction error common to all compartmentalized models, an error that is independent of model parameters.

When we removed this reaction from the models, we found that it did not affect the predictions for the high correlation models, and improved all remaining correlations to >0.9, with the exception of the Yeast 4 model, which still over-predicted the maximum biomass flux at high glucose:oxygen exchange constraint ratios (Fig 6C).

### 5) Predictive ability for single-gene essentiality did not correlate with predictive ability for our reference list of pairwise synthetic lethal genes

**Fig 7:**
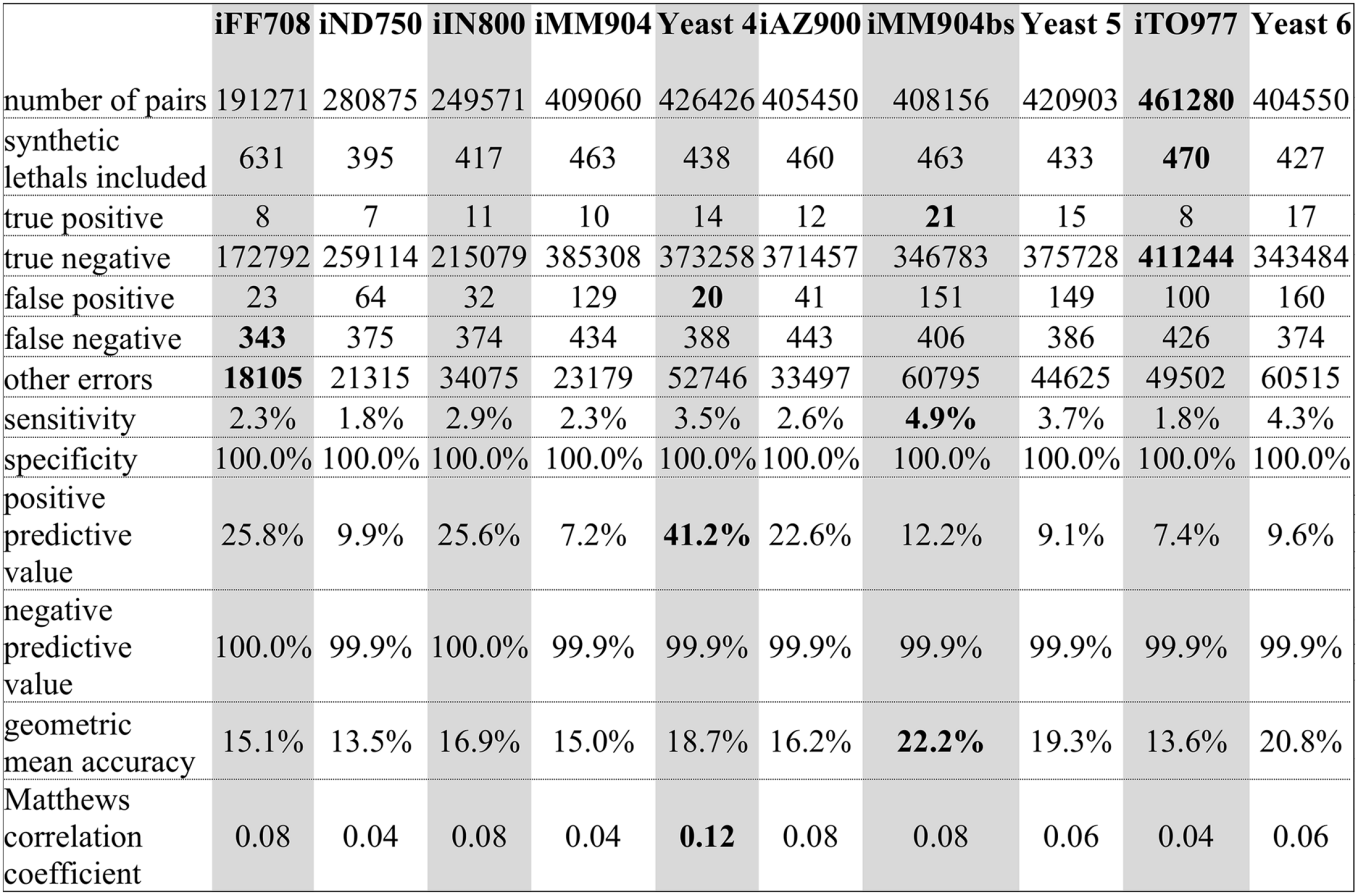
Double knockout simulation results. Summary statistics of our screen of double gene deletions for synthetic lethal gene pairs. Highest values for each statistic are in bold. "other errors" includes the false prediction that either gene is individually essential.

Using the models as distributed (i.e., with tuned biomass definitions and default exchange reaction constraints), we conducted a simulated screen of all pairwise deletions for 10 models (iFF708, iND750, iIN800, iMM904, Yeast 4, iAZ900, iMM904bs, Yeast 5, iTO977, and Yeast 6). Using a strict definition of synthetic lethality in which neither gene is individually essential, but are pairwise essential for growth, we found that the MCC for model prediction of synthetic lethal gene pairs ranged from 0.04 to 0.12, when compared to a list of synthetic lethal gene pairs that we generated using the Saccharomyces Genome Database Yeastmine tool [52] (Fig 1). Additional summary statistics of these screens are included in Fig 7.

Surprisingly, we found that the MCC for synthetic lethal interactions did not correlate with the MCC for single-gene essentiality (R^2^ = 0.0253). The relatively low predictive ability of these metabolic network models for synthetic lethal gene pairs may be attributed in part to the fact that the reference list of synthetic lethal gene pairs is not well-established due to the challenge of conducting pairwise gene deletion screens *in vivo* [53], the fact that predictions of multiple perturbations to a genetic network require more complex analysis [54], and synthetic lethality phenotype observations may be greatly influenced by experimental design [16]. We anticipate that evaluating and improving constraint-based phenotypic predictions of multiple-gene deletions will advance hand in hand with efforts to experimentally explore gene interaction networks.

The source code used for conducting these simulations is included as S1 File. Detailed results of all simulations conducted, including lists of true and false predictions for each model in each simulated single-knockout screen, are attached as S2 File.

## Discussion

The main findings of this study are that the iterative publication of many models over the past two decades has generally, but not universally, improved yeast metabolic network reconstructions when assessed by a range of metrics. We also found that current approaches to model development and annotation can hinder direct assessment of the underlying reconstruction. Thus, this study serves to provide an overview of the historical development of yeast metabolic network models over the past two decades, provide methods for evaluating future metabolic models, and highlight opportunities for improving the reconstruction of the yeast metabolic network. It also raises issues that should be considered so that metabolic reconstruction efforts can best contribute to investigations of metabolic processes.

### Yeast metabolic network model performance varies according to evaluation metrics

When we directly compare functional models of the yeast metabolic network published over the past two decades, we note generally increasing trends in genomic coverage (using either verified or total number of open reading frames annotating model reactions), number of metabolites, and reactions. We do not discern strong trends in number of dead-end metabolites in models, the percentages of reactions associated with genes, or in predictive accuracy for synthetic lethal genetic interactions. When comparing flux balance predictions with model default or standardized simulated media, objective functions, and reference lists of “essential” genes, we find that predictive power for single-gene essentiality has gradually improved. However, the observed trends are not uniform across all models and simulations we conducted. Each model has its own strengths and weaknesses; as demonstrated in a previous comparative study, “different models may be preferable for use in different applications” [44].

The uneven progress in improving model performance metrics reflects the historical path of iterative model refinement. Different models are developed to address different research questions, and are not necessarily focused on improving gene essentiality predictive accuracy. Thus, each new model may advance (or regress) when compared to previous models depending on the metrics used to assess the model. This is particularly evident when examining the relative performance of single gene essentiality predictions (Fig 3). For example, iND750 greatly expanded compartmentalization in the yeast metabolic reconstruction, but had lower predictive ability for single-gene essentiality than the earlier iFF708 model in our analysis. Development of the iAZ900 model demonstrated the utility of optimization-based procedures for improving model prediction, so it has the highest MCC for the reference conditions used for model development, but not the highest overall MCC across all conditions. The iIN800 model expanded the reconstruction of lipid metabolism, and the iTO977 model expanded the scope of yeast metabolic models to facilitate transcriptomic analysis. As new models integrate and improve upon earlier models, a path dependency on previous modeling or reconstruction efforts emerges. This necessary relationship can lead to iterative improvement, but can also propagate errors and complicate assessment of the reconstruction of the yeast metabolic network. Further, as models have been developed, different research groups have used different tools to validate their models (such as different lists of genes reported to be essential in a particular strain background or experimental condition). Thus, no model should be considered “best” or definitive for all applications. Examining simulations across multiple models may be a prudent approach for building confidence in predictions.

The results of our comparison of predicted maximum achievable biomass flux to measured growth rates emphasize that model users must take great care when imposing multiple constraints prior to conducting FBA, or when interpreting experimental growth rate measurements as being attributable to a single limiting nutrient. If a model user is attempting to compare simulated predictions with observed growth rates that scale linearly with the concentration of a single limiting nutrient, our results suggest that they should test to ensure that the model is operating within a linear range in which the desired nutrient is in fact the sole factor limiting predicted flux to the objective. If operating within such a regime, users could confidently scale FBA results by either varying model parameters (such as ATP maintenance demands) or by simple linear transformation of objective values found via FBA. However, model users must be wary of discontinuities arising from shifts in the limiting nutrient (such as from glucose to oxygen).

The high correlation between predicted biomass flux and observed growth rates in high glucose:oxygen exchange constraint ratio regimes is surprising not only because “normal yeast mitochondrial structures are disrupted when glucose levels are high” [55], but because c. 2,000 genes are regulated by the diauxic shift [56]. These changes are dependent upon concentration, rather than flux [57]. Thus, it is likely that there are sets of constraints that should be applied to a metabolic network for condition-specific modeling. We did not observe that such constraints were necessary for predicting the stoichiometrically-determined shift from glucose-limited to oxygen-limited maximal growth rate over a range of glucose to oxygen exchange flux ratios. Differentiating universal constraints (such as chemical stoichiometry) from condition-specific constraints appears to have great potential as a fruitful avenue for future research efforts.

### Current approaches to model development and annotation hinder direct assessment of reconstruction efforts

We found that model gene essentiality predictions are biased by factors that are not reflective of the accuracy or completeness of the metabolic network reconstruction. Such factors include reference gene lists, choice of objective function for flux balance analysis, and simulated media used for in silico screens. However, it is likely that standardizing these factors (as we have done in this study) for comparing models is not sufficient for assessing the quality of the metabolic network reconstruction; model builders must make other choices when developing a model that is amenable to simulation from a network reconstruction. For example, since different models use different approaches to fill gaps in the known metabolic network or to ascribe catalytic function to a poorly characterized yeast genes, different models are likely to include different hypothetical transport or biochemical reactions with different levels of evidence or confidence in the accuracy of the functional role of a protein.

Reconstructing a metabolic network provides an opportunity to highlight areas of uncertainty to productively guide future research efforts. This opportunity is distinct from the utility of mathematical simulation of fluxes using metabolic network models. In fact, deriving a metabolic network model from a reconstruction can obscure the knowledge gaps or uncertainty that can be highlighted during the process of network reconstruction [7]. This risk is particularly acute where poorly understood portions of metabolism are not clearly implicated in the research to which a model is being applied, or when highlighting knowledge gaps or ambiguity may hurt model performance according to metrics used to assess predictive performance or scope. For the yeast models we compared, some of which use current annotation and model exchange protocols and formats, there is no mechanism for a model user to identify knowledge limitations discovered during reconstruction of the underlying network, nor is there sufficient annotation describing the specific techniques used to address such limitations when the model was constructed within the published model itself.

The current state-of-the-art for metabolic network modeling presents a significant barrier to entry for researchers who are not familiar with the idiosyncrasies of each model because these idiosyncrasies are not sufficiently documented within the model structure itself. Thus, though we observed that model predictions of gene essentiality are generally better for models evaluated with a simulated medium containing glucose as the primary carbon source than model predictions when using ethanol, glycerol, or galactose as a carbon source, we cannot conclusively attribute the improvement in glucose-essential prediction to improvements in the reconstruction of the biochemical reaction network because there is no clear mechanism for separating the information contained in the underlying reaction network reconstruction from the modeling assumptions and choices made in deriving a particular metabolic network model. Similarly, we cannot conclusively attribute the relative lack of improvement in predictions with non-glucose carbon sources among models to errors in the reconstruction rather than faulty model assumptions, idiosyncratic objective function definition (i.e., model overfitting), or biological factors such as condition-dependent gene essentiality for genes included in the reference list of “essential” genes.

Selecting appropriate data sets for model validation presents an additional challenge to the reconstruction effort. Specifically defining the media and conditions in which a given gene (or combination of genes) is essential remains an ongoing and important area of research to advance our understanding of metabolism. In the absence of well-defined reference phenotypes, we cannot confidently ascribe the low predictive ability for pairwise essentiality to errors in metabolic network reconstruction, uncertainties in synthetic lethal phenotypes, or physiological processes which are not metabolic, such as gene regulation or cell cycle checkpoint events. Further evaluating and improving model predictive performance for conditional essentiality will be greatly assisted by use of new prototrophic yeast strains and genetic screens in specifically designed media [46].

Despite these methodological challenges, there is benefit to comparing metabolic network models for the same organism for filling gaps and for identifying mistakes and opportunities for further expansion of the metabolic network reconstruction. We note, for example, that iron metabolism is important to mitochondrial function, but is not included in these models. None of the models include folate, chitin, or hypusine in the biomass definition, a model building choice that leads to false negative gene essentiality predictions and dead-end metabolites, but also highlights opportunities for expanding the reconstruction of the yeast metabolic network. Similarly, since most models have been validated with laboratory results from strains originally designed to facilitate genetic investigation (strains which bear auxotrophic markers in their genetic backgrounds) [48], it is likely that the reconstruction of portions of the yeast metabolic network (such as nitrogen and sulphur metabolism) is incomplete. Updating the reconstruction in support of research with a new prototrophic yeast mutant library [46] provides an exciting opportunity for refining our understanding of yeast metabolism.

As different groups refine yeast metabolic network reconstructions and models, there should be a convergence to a full, accurate reconstruction of the complete network. We do not observe evidence that supports marked changes in the reconstruction, such of a marked shift in model predictive ability or genomic coverage in our analysis of models published to date. Further, recent work has observed that many enzymatic functions are not included in existing models and reconstructions [8]. Thus, the effort to reconstruct the yeast metabolic network is incomplete. Increased efforts to expand the scope of reconstruction, such as including signaling and regulatory network processes, may provide a way to advance efforts to reconstruct organism-specific networks.

Our analysis suggests that metabolic network reconstruction efforts could benefit from emphasizing the distinction between reconstruction of known or hypothesized metabolic function, and metabolic models developed for particular applications. A reconstruction may be improved, but model performance may drop by some metrics (for example, adding a parallel metabolic pathway could lead to false negative gene essentiality predictions in the absence of regulatory constraints blocking an available network branch, or conversely, adding condition-specific regulatory constraints could hinder predictive value for conditionally-essential genes in other environments). Similarly, model performance could be enhanced in some cases by removing established biochemistry (and such a choice would be defensible if modeling a particular environment in which a portion of the metabolic network was unavailable due to regulation). Thus, we find that no single metric we used to compare metabolic network models is sufficient to evaluate the progress of the yeast reconstruction efforts. Models should be assessed by gene essentiality predictions, as well as the extent of evidence and annotation for included information, the size of the network, and network connectivity metrics. Unfortunately, current methods for annotating the workflow of model development makes such analysis challenging. In some cases, erroneous model predictions have been computationally corrected through changes that cannot be annotated in exchange formats. Thus, they become obscured, rather than highlighted in a way that would better facilitate further investigation. Similarly, although great efforts have been expended to assess the evidence for information in the published models, none of the SBML files we evaluated included confidence scores or full annotation of literature sources, so these assessments remain internal to a development group and are not effectively propagated to subsequent model users. This is in part a historical artifact—many existing standards such as SBML are intended to distribute models, rather than fully annotated reconstructions. Efforts such as the definition of the Pathway Tools schema [58] lay important ground work towards broader community participation in improving the process of metabolic network reconstruction and metabolic model derivation. Though reconstructing metabolic networks has been the focus of biochemistry for more than a century, computational metabolic network reconstruction is still a young field with great contributions to make. Through this comparative analysis of yeast metabolic network models, we hope to contribute to the ongoing efforts to improve our understanding of metabolism through collaborative network reconstruction, and to highlight opportunities for improving the process of metabolic network reconstruction and model derivation.

## Materials and Methods

All simulations were conducted on a laptop running Windows 7 (Microsoft) using MATLAB 2013a (MathWorks Corporation, Natick, Massachusetts, USA), with SBML Toolbox version 4.1.0 [59], COBRA Toolbox version 2.05 [60], and Gurobi Optimizer version 5.6 (Gurobi Optimization, Inc., Houston, Texas, USA). All code written for this study is included as S1 File.

### Models

Models were obtained from public repositories, supplemental information, or research collaborators and modified as follows:

- iFF708—SBML file downloaded from the BioMet Toolbox [61] at http://129.16.106.142/models.php?c=S.cerevisiae. SBML file modified by KS's make_models_consistent script (included) as follows: modify SBML namespace; add “_b” to boundary metabolites to accommodate COBRA Toolbox convention;
- iND750—downloaded from BIGG database [62] at http://bigg.ucsd.edu
- iIN800—provided by Markus Herrgard as a.mat file; differs from published version to enable FBA with COBRA Toolbox
- iMM904—downloaded from BIGG database [62]
- Yeast 4—downloaded from yeast.sf.net
- iAZ900—from [20] supplemental info
- iMM904bs—[32] http://www.utoronto.ca/boonelab/data/szappanos/
- Yeast 5—downloaded from yeast.sf.net
- iTO977—provided by Markus Herrgard—differs from published version with addition of CoA sink to enable FBA with the COBRA Toolbox. Modifications include: setting objective function, adding reaction-gene annotation to Herrgard version from published version, add ChEBI metabolite identifiers from supplemental data to model structure, copy KEGG IDs from Yeast 7 model using common ChEBI identifiers.
- Yeast 6—downloaded from yeast.sf.net
- Yeast 7—downloaded from yeast.sf.net
- Biomodels.db—generated by path2models software [6], downloaded 11/21/13 from biomodels database [63] at https://www.ebi.ac.uk/biomodels-main/BMID000000141353

Each of the models is provided in S1 File, formatted as.mat files containing the COBRA Toolbox data structure with any modifications to enable simulation.

### Reference gene lists for knockout growth/no-growth evaluations

Different sets of genes have been observed to be essential for growth in different conditions, and different lists have been used for previous evaluations of model predictive accuracy. Therefore, we generated our own reference lists for the current comparative analysis. We began with the list of 1,120 unique open reading frames annotated as essential by the Yeast Deletion Project (available at http://www-sequence.stanford.edu/group/yeast_deletion_project/downloads.html), then removed YCL004W and YKL192C, based upon literature review [64,65]. Since this list was generated from experiments using a complete medium, we used it as our reference for simulations of growth in a synthetic complete medium (our approach to defining simulated medium is described in the “Flux Balance Analysis—Medium Definition” section below).

For evaluating simulations of growth in a glucose-limited minimal medium, we supplemented the essential gene list with 441 additional open reading frames reported to induce auxotrophy upon deletion. This list was generated by downloading a list of ORFs annotated as auxotroph-inducing in the Saccharomyces Genome Database, removing those already included in the essential list and temperature-sensitive inositol auxotrophs, and modifying the list based on literature-based curation as described in the testYeastModel.m file, which is included in S1 File. Combining the essential ORF list with the auxotroph-inducing list resulted in a list of 1560 open reading frames as our reference list of genes considered essential in a minimal medium.

We also compiled reference lists of essential genes based on the screen of non-essential genes in the knockout collection in defined media with different carbon sources conducted by Kuepfer et al. [17]. This screen evaluated 4869 open reading frames included in the yeast knockout collection. Applying the stringent standard of a score of 0 for ORF essentiality, we classified 59 ORFs as essential in defined medium with glucose as the sole carbon source, 307 with galactose, 291 with glycerol, and 332 with ethanol. When comparing model predictions for these medium, we did not evaluate all ORFs in each model, but instead only characterized the subset of genes in the model that were also evaluated in the Kuepfer et al. screen. These lists of essential genes are included in the testYeastModel_kuepfer.m script, which is included in S1 File.

We built a reference list of synthetic lethal genetic interactions (pairs of ORFs that are not individually essential, but become essential when both are deleted) by querying the Saccharomyces Genome Database with YeastMine [52]. We built an XML query to search for interacting genes where the experiment type was listed as “Synthetic Lethality”. The resulting report was downloaded and imported to MATLAB to generate a list of 32,488 pairs of ORFs that have been annotated as synthetic lethal. The specific XML query is described in the analyze_double_results.m script, which is included in S1 File.

We note that any reference list of gene essentiality is dependent upon experimental conditions, so different researchers may construct such lists in different ways. The predictive accuracy of any model is a function of the standard used, so different reference lists are expected to affect the specific MCC value of its agreement with observation. Thus, it is particularly important that the same list of “essential” genes or gene pairs be used when comparing different models.

### Flux balance analysis—medium definition

We conducted flux balance analysis of each model using both the model-default simulated medium composition, along with media formulations we defined in an effort to standardize model predictions. We defined the following media for our simulations: a minimal medium that enabled predicted biomass production for all the models in which glucose is the sole carbon source; a synthetic complete medium with glucose as the sole carbon source, which was based on previous computational screening efforts [66]; and the synthetic medium defined by Kuepfer et al., using glucose, galactose, glycerol, or ethanol as the sole carbon source.

The minimal medium was simulated by allowing unconstrained exchange of ammonia/um, oxygen, phosphate, sulphate, and setting a constrained uptake of glucose. The iMM904bs and Biomodels.db models did not predict growth using this medium when using their default biomass definitions (biomass definitions are described below in the “Flux Balance Analysis—Biomass Definition” section). To enable FBA, the simulated minimal medium for these models was supplemented: the iMM904bs model required iron exchange, and the Biomodels.db model required the amino acids L-tyrosine, L-lysine, L-isoleucine, L-arginine, L-histidine, L-methionine, and L-tryptophan.

The code used for setting the medium for each model is included in S1 File in the testYeastModel.m and testYeastModel_kupefer.m scripts.

### Flux balance analysis—biomass definition

We conducted FBA for all models except the Biomodels.db model with two different objective functions: first, we used the model’s default biomass definition, as included in the published version of the model; second, we used a common biomass definition as similar to the iFF708 biomass definition as each model’s exchange reactions and metabolites allowed. The Biomodels.db model did not predict growth with the iFF708 biomass definition, so we only used its default objective function to verify functionality. We selected the iFF708 biomass definition as a reference standard because it was the objective function that most models could satisfy. For example, iFF708 would not be able to satisfy the Yeast 5 biomass definition due to the expanded sphingolipid requirement in the latter. Our use of the common, older biomass definition was intended in part to separate model improvements that arise from improved reconstruction from those that arise due to a more specific biomass definition.

The code used to set the biomass definitions for each model is included in S1 File in the testYeastModel.m and testYeastModel_kupefer.m scripts.

### Flux balance analysis—analyzing mutant phenotypes and evaluating model predictions

Accounting for the seven media compositions and two biomass definitions described above, we conducted flux balance analysis to predict single-gene deletion growth phenotypes for each model in fourteen different conditions. We elected to use used a tight threshold of binary growth/no growth prediction when comparing model growth predictions to our reference lists of essential genes because flux balance analysis of metabolic network models may be less predictive for mutant growth rates than for a binary essential/non-essential gene classification [46] and because growth rate predictions may be tuned by adjusting model parameters such as ATP maintenance reaction demands or constraints on carbon source utilization reactions. For this study, a gene was considered to be predicted as essential only if flux balance analysis of a simulated mutant predicted a maximum flux to the biomass objective of less than 1 x 10^−6^ flux units.

The agreement between model gene essentiality predictions and the reference lists was quantified using the Matthews’ Correlation Coefficient (MCC) (eq 1) [67], a metric that considers true positive, true negative, false positive, and false negative predictions without any assumption of the frequency of observations in the reference dataset. MCC ranges from -1 (when model predictions are the exact opposite of the reference dataset) to +1 (when model predictions match the reference data set).

$$MCC=\;\frac{TP\times TN-FP\times FN}{\sqrt{\left(TP+FP\right)\left(TP+FN\right)\left(TN+FP\right)\left(TN+FN\right)}}$$ (1)

Where true positives (TP), true negatives (TN), false positives (FP), and false negatives (FN) are defined as in [17]: a true positive prediction is one in which the model predicts that a gene is not essential for biomass production, and the gene has been annotated as not essential. Values for each confusion matrix, along with lists of positive and negative predictions, are included as S2 File.

We also assessed model prediction of synthetic lethality, or double-gene deletion phenotypes, for 10 of the models. When comparing model predictions to the reference gene list, we defined true positive as predictions in which neither gene in a reported synthetic lethal pair is predicted to be essential by itself, and the pair is essential. We defined true negatives as predictions in which neither gene is predicted to be essential by itself, the pair is not predicted to be essential, and the pair is not reported to be synthetic lethal. We defined false positives as predictions in which neither gene is individually predicted to be essential for growth and the pair is predicted to be essential for growth, but the pair has not been reported to have a synthetic lethal interaction. We defined false negative as predictions in which neither gene is individually predicted to be essential and the pair is predicted to be non-essential, though a synthetic lethal interaction has been reported. We categorized incorrect predictions of single-ORF essentiality as “other errors”–such errors were not included in our MCC calculation, since they were accounted for in the in silico single gene knockout screen. We did not modify the models’ biomass definition or simulated medium composition for our double knockout simulation. We also note that our definition of synthetic lethal interactions, which requires a model prediction of greater than 10% of the predicted wild-type biomass flux, is an arbitrary, but strict requirement. It is likely that the MCC for synthetic lethal predictions would be influenced by the choice of minimum biomass flux, and we selected 10% as a representative example for this particular analysis. If slow-growing double mutants are scored as synthetic lethal in an in vivo screen, and included in our reference list of synthetic lethal pairs, a correct model prediction of low biomass flux could be scored as false negative.

### Model network structure—blocked reactions and dead-end metabolites

Blocked reactions are reactions that cannot carry a flux in a given simulation condition; thus, the number of blocked reactions may change for a given model with different biomass definitions or different allowed exchange reactions. We used the fastFVA module [68] to count the number of blocked reactions for each model when all exchange reactions were allowed to carry flux, and using both the model default and the iFF708 biomass definitions.

Dead-end metabolites are metabolites that either participate in only one reaction, or can only be produced or consumed. Thus, they are a network feature that is not influenced by exchange reaction or biomass definition changes. We counted the number of dead-end metabolites in each model with the COBRA Toolbox detectDeadEnds function.

The code used for blocked reaction and dead-end metabolite analysis is provided in S1 File. Model-specific lists of blocked reactions and dead end metabolites are included as S2 Table.

### Model scope comparison

Similarity of genomic coverage among models was assessed by hierarchical clustering based on pairwise distance of binary vectors of logical values for open reading frames included in a model’s reaction annotation (i.e., 1 if a given ORF is included in a model, or 0 otherwise). The binary vectors are presented as a heat map, and clusters are presented as a clustergram and scatterplot (generated with classical multidementional scaling) in Fig 2.

Different model developers have annotated metabolites in different ways, so we began our comparison of metabolites by expanding the annotation of models by adding identifiers from the Chemical Entities of Biological Interest (ChEBI) database [69] to metabolites where possible. We were able to establish ChEBI annotation for different subsets of metabolites in each model, so this comparison is, by necessity, less comprehensive than comparison of model genomic coverage. The Biomodels.db model annotates metabolites with multiple ChEBI identifiers (reflecting redundancy in the ChEBI database). We chose the first ChEBI identifier when comparing the Biomodels.db model with models derived from manual reconstruction. Other models did not include multiple ChEBI identifiers for annotated metabolites. Like genomic coverage, metabolic coverage was scored with a binary vector of logical values, and the comparison is presented as a heatmap, clustergram, and scatterplot.

A sorted list of genes by models and all code used for scope comparison are included as Supporting Information.

### Evaluating model predictions of maximum biomass flux

We compared predictions made by media- and objective-normalized models with the aerobic growth rates reported by Österlund et. al. [37] for “N-limited” and “C-limited” conditions (we did not simulate anaerobic growth since most of the models we are examining do not predict anaerobic growth on a minimal medium). We conducted flux balance analysis of each model after standardizing model objective functions to the iFF708 biomass objective, and then applying constraints to the glucose, oxygen, and nitrogen exchange fluxes, first individually and then in combination. We used the measured uptake values reported by Österlund et al. [37] as constraints for each of these exchange reactions.

## Supporting Information

S1 Table Comparison of model genomic coverage. Genes are row-aligned to facilitate comparison of model genomic coverage.
(XLSX)

S2 Table Blocked reactions and dead end metabolites. Blocked reactions (listed by model reaction ID) and dead end metabolites (listed by metabolite ID) were identified using the code in S1 File.
(XLSX)

S3 Table Growth rate simulation results. Maximum achievable biomass flux was compared to growth rates reported by Österlund et al. using code in S1 File.
(XLSX)

S1 File Source code for model simulations. Code used for comparative analysis of yeast metabolic network models. The FBA tests folder includes the scripts used to specify model medium, biomass definitions, and essential gene lists for FBA-based comparison of gene essentiality predictive accuracy for single and double gene deletions. These scripts can be run with the runanalysis.m script. The doubles folder includes the scripts and results of the double gene deletion simulations. The models folder includes matlab.mat files of the models. The \models_as_used subfolder includes models with any modifications used for this analysis. The scope comparison folder includes the code used to compare metabolite and gene scope for the models.
(ZIP)

S2 File Detailed single knockout screen results by model. This folder includes the output of all single knockout simulations conducted, including predictions considered true positive, true negative, false positive, and false negative for each model analyzed.
(ZIP)
